# Supplementary material for: Landscape and climatic features drive genetic differentiation processes in a South American coastal plant
Source: BMC Ecol Evol. 2021 Oct 26;21:196. doi: 10.1186/s12862-021-01916-4 (PMC8547116; doi:10.1186/s12862-021-01916-4)
Supplement: Supplementary file 1 — Additional file 1: Figure S1. Observed vs. expected heterozygosity for each locus. Figure S2. (A) Plots of the best K estimates for Structure results. (B) Plots of the mean and standard deviation of the deviance information criterion (DIC) obtained for each maxK assessed with TESS. Figure S3. Bar plots of the individual membership for each genetic cluster obtained with Structure. White dashed lines separate populations, and names are indicated on the figure top side. Figure S4. The plot of Bayesian information criterion (BIC) values obtained for each K number assessed using the multivariate method Discriminant Analyses of Principal Components. Figure S5. Graphical representation of the four coalescent migration models tested in Migrate-N for Calibrachoa heterophylla. (A) Source-sink from inland; (B) Source-sink from the west; (C) Step-stone from inland; (D) Step-stone from coast. Figure S6. Graphical representation of the raster layers used to calculate the connectivity values in topographic tests (A) Continuous model; (B) Water bodies model. Table S1. Migration estimates obtained with three independent runs of BAYESASS. The values indicate the estimated posterior mean effective migration rate per generation [the fraction of individuals in population i (rows) that are migrants derived from population j (columns)], and the numbers in parentheses show the standard deviation. Bold values indicate the diagonal (intra-population estimates), and red values indicate the highest migration estimates (those with above zero 95% confidence intervals). [file 12862_2021_1916_MOESM1_ESM.pdf]

Figure S1. Observed vs. expected heterozygosity for each locus.

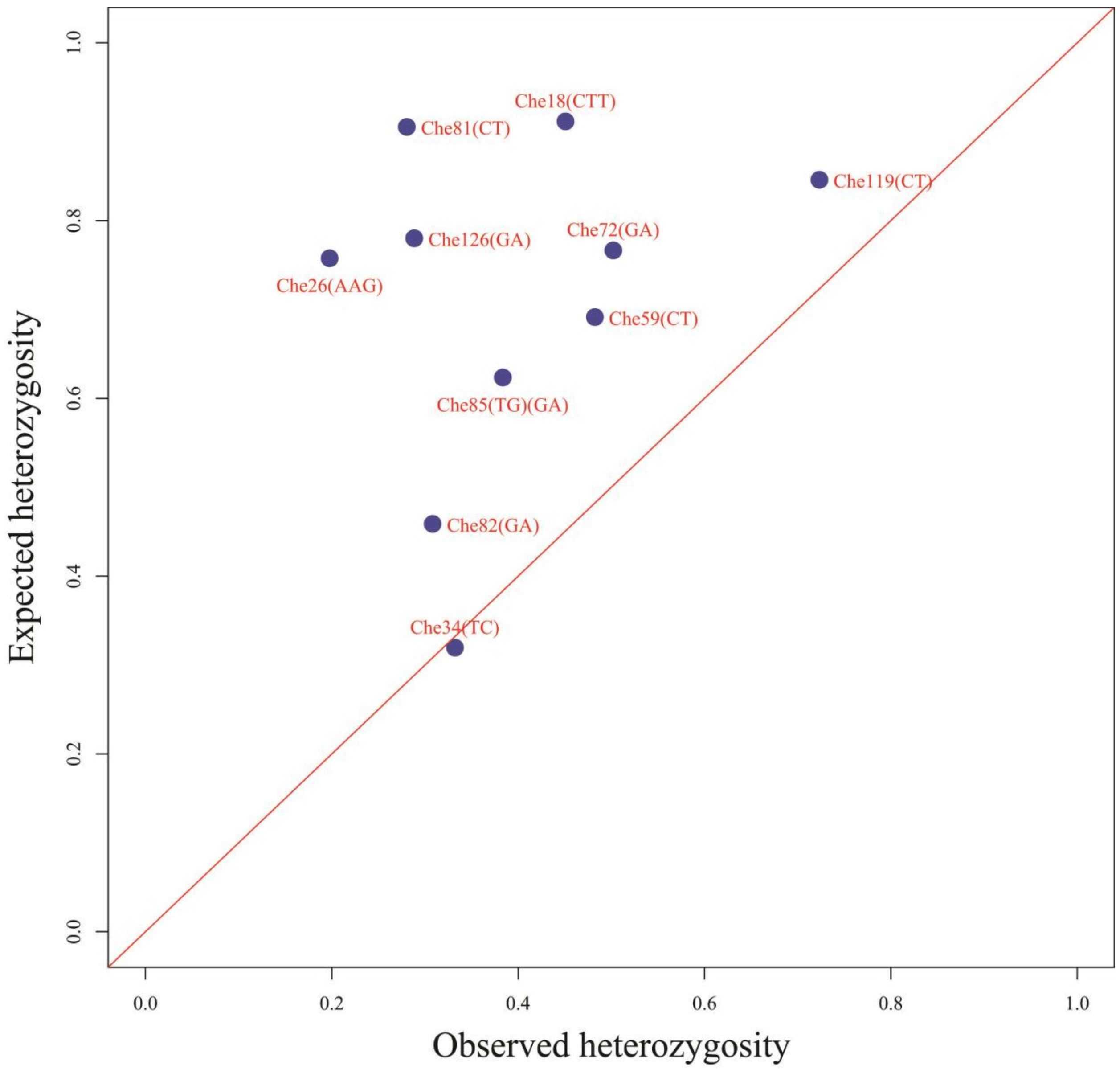

Figure S2. A. Plots of the best K estimates for Structure results. **B.** Plots of the mean and standard deviation of the deviance information criterion (DIC) obtained for each maxK assessed with TESS.

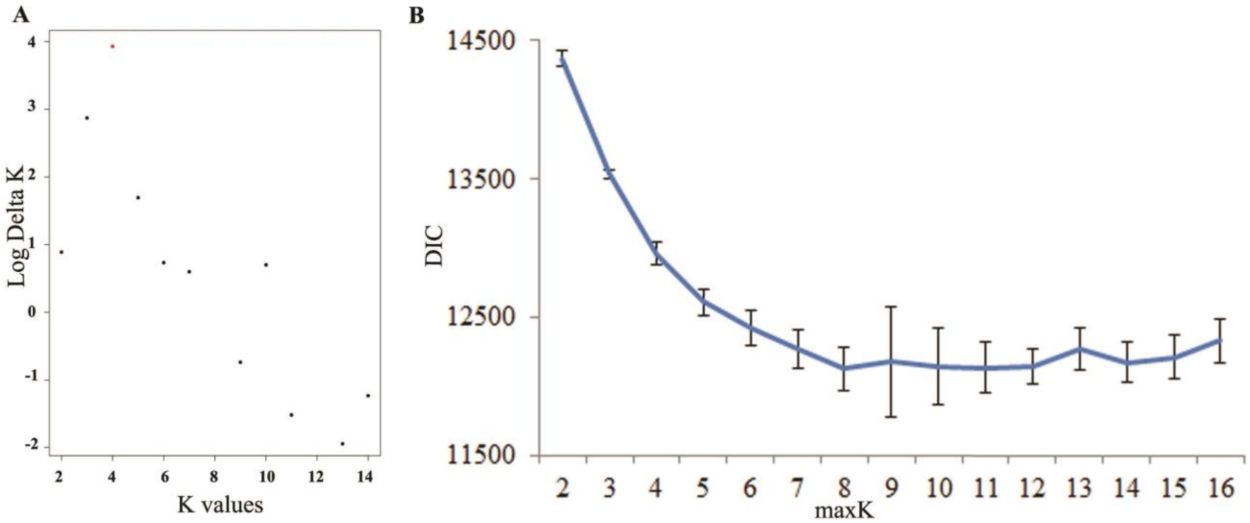

Figure S3. Bar plots of the individual membership for each genetic cluster obtained with Structure. White dashed lines separate populations, and names are indicated on the figure top side.

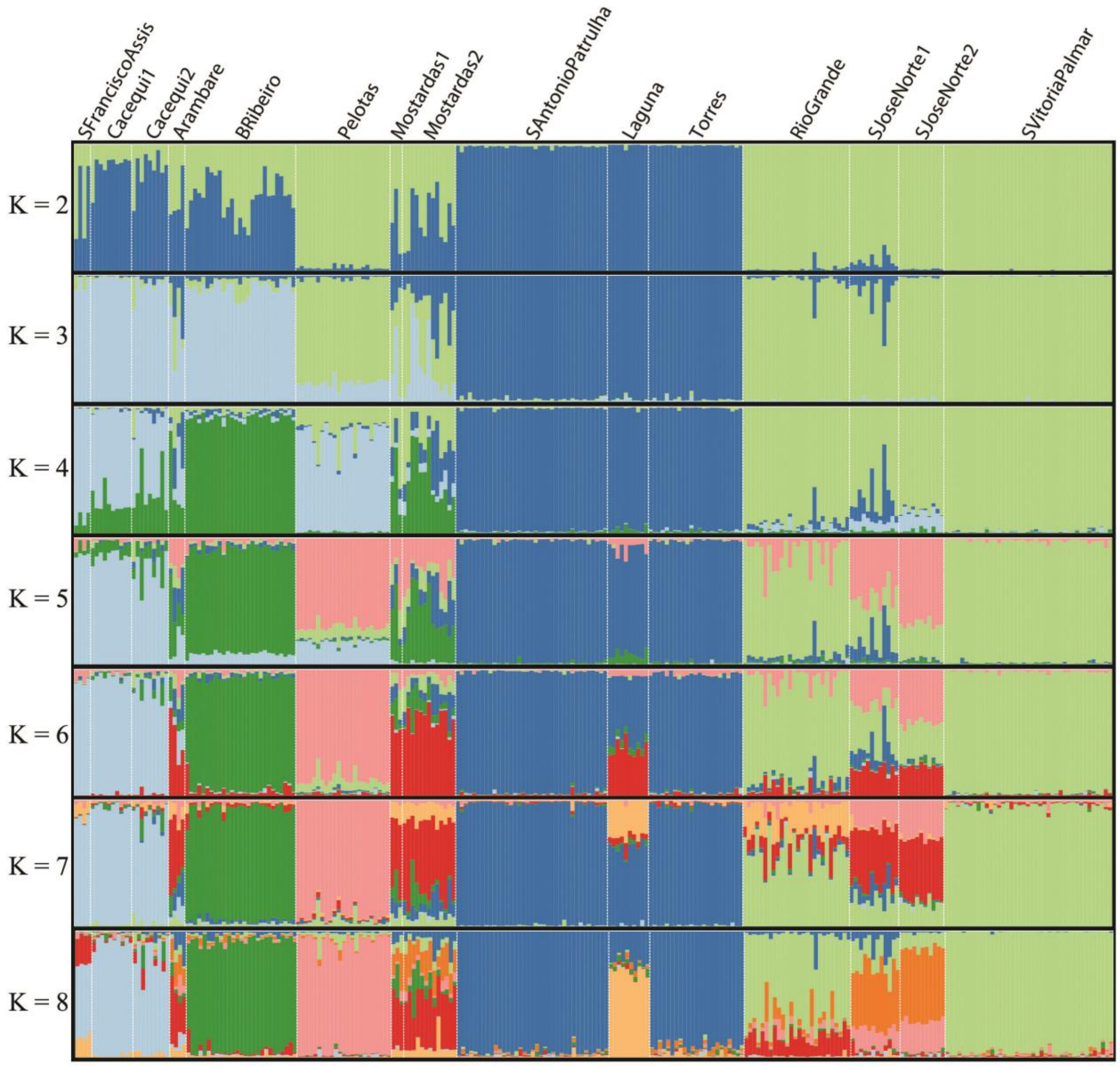

Figure S4. The plot of Bayesian information criterion (BIC) values obtained for each K number assessed using the multivariate method Discriminant Analyses of Principal Components.

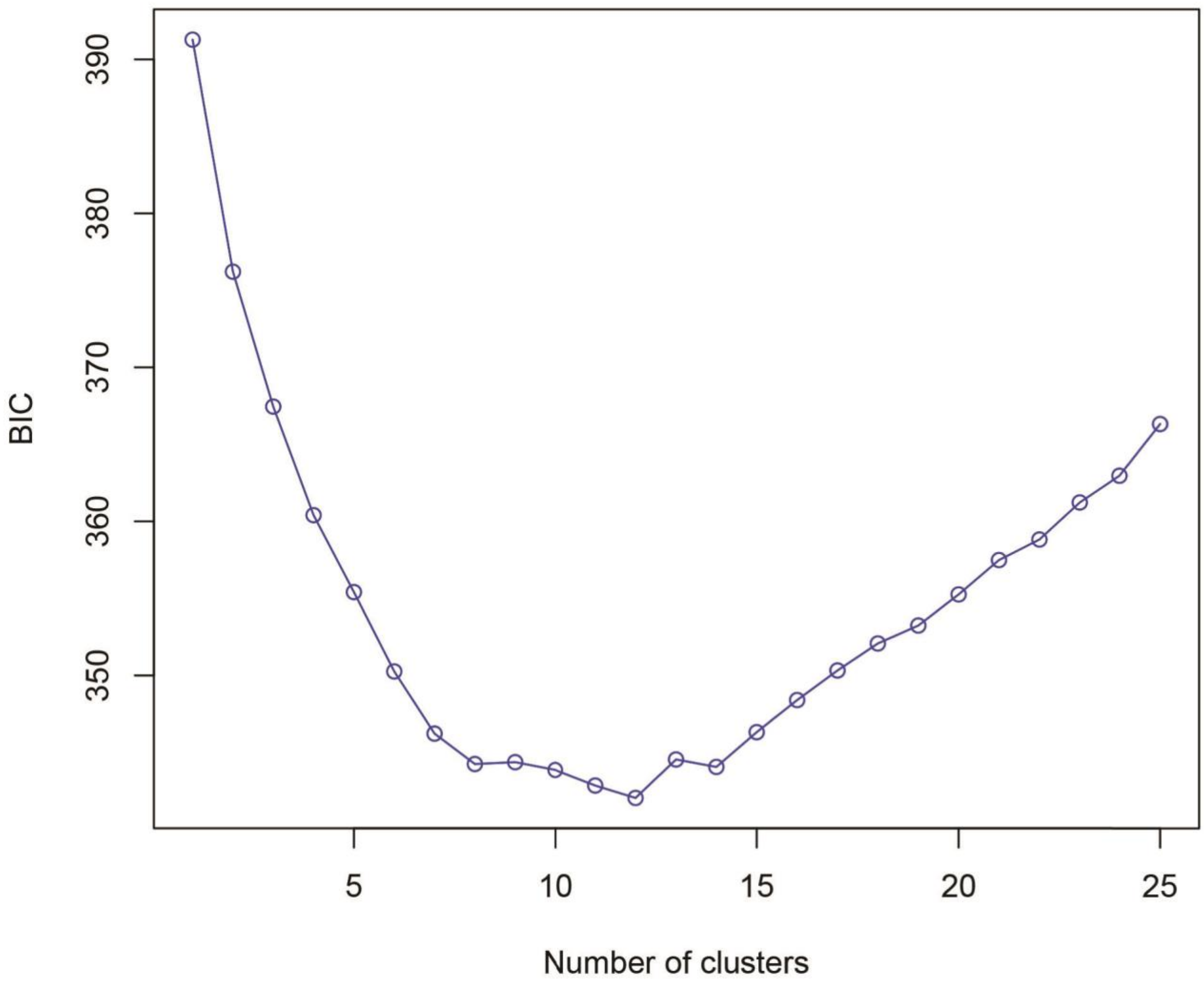

Figure S5. Graphical representation of the four coalescent migration models tested in Migrate-N for Calibrachoa heterophylla. **A.** *Source-sink from inland*; **B.** *Source-sink from the west*; **C.** *Step-stone from inland*; **D.** *Step-stone from coast*.

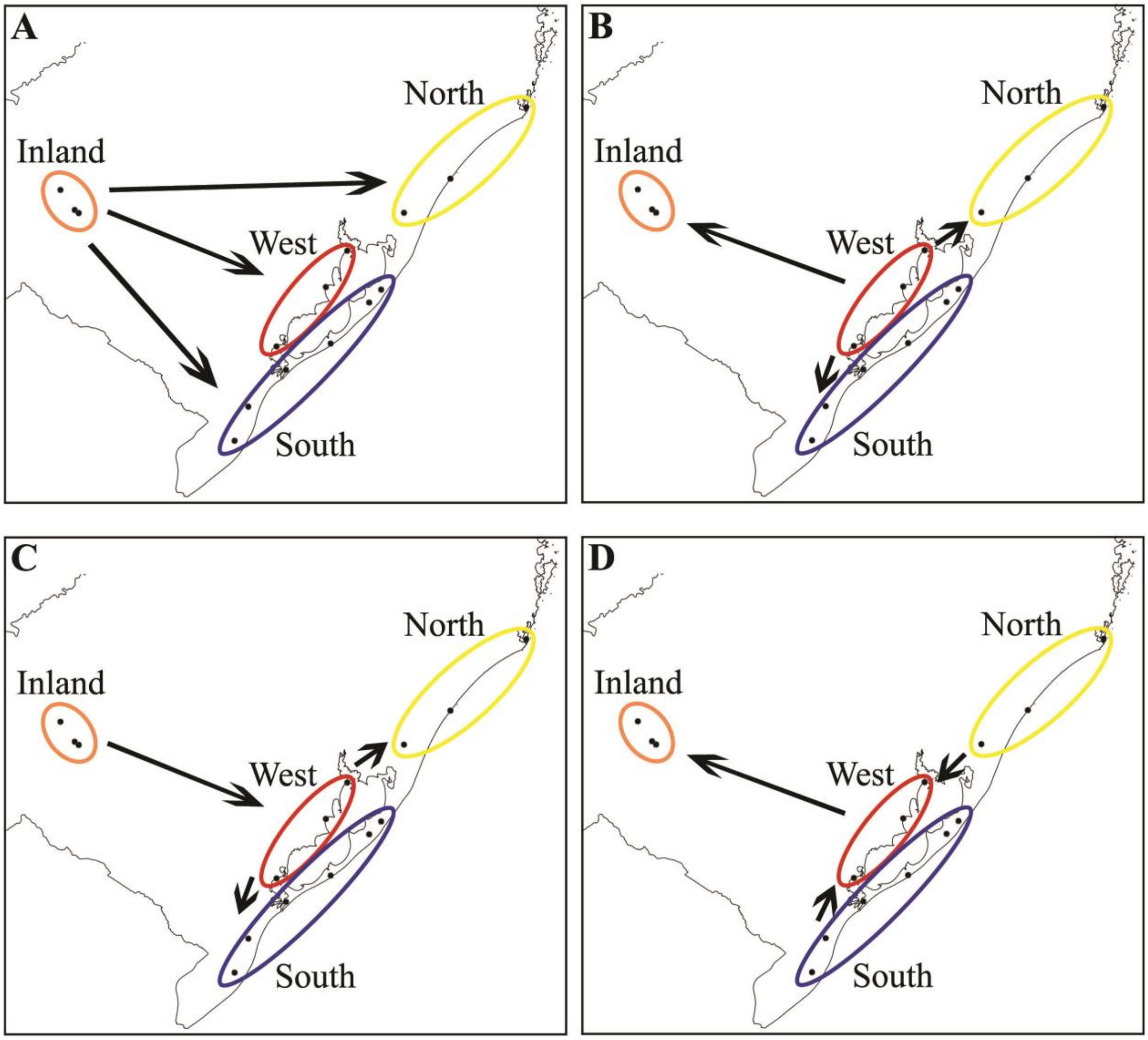

Figure S6. Graphical representation of the raster layers used to calculate the connectivity values in topographic tests **A.** *Continuous model*; **B.** *Water bodies model*.

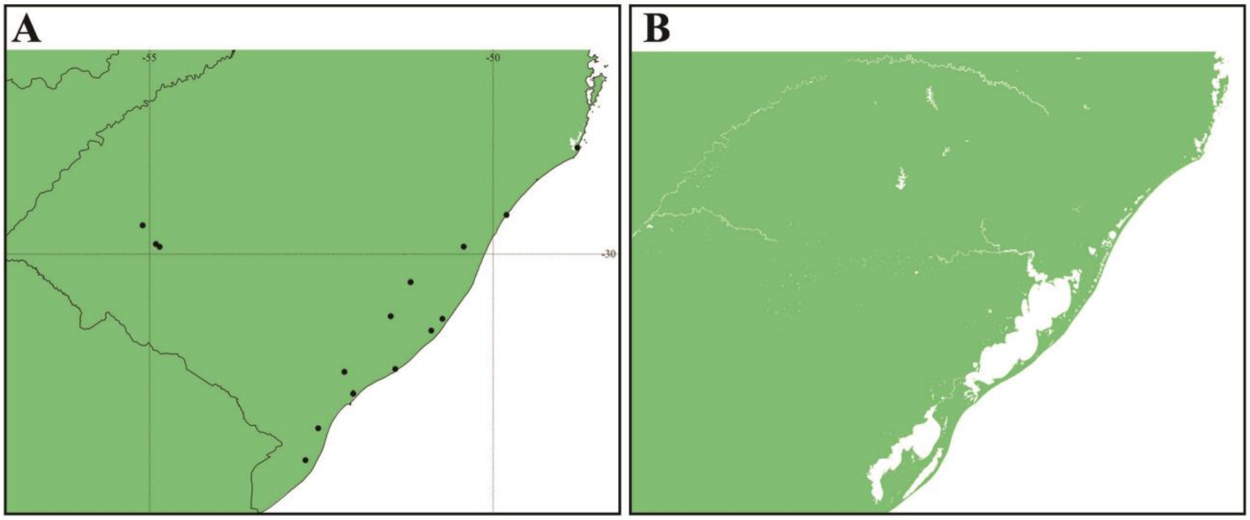

Table S1. Migration estimates obtained with three independent runs of BAYESASS. The values indicate the estimated posterior mean effective migration rate per generation [the fraction of individuals in population *i* (rows) that are migrants derived from population *j (*columns)], and the numbers in parentheses show the standard deviation. Bold values indicate the diagonal (intra-population estimates), and red values indicate the highest migration estimates (those with above zero 95% confidence intervals).

**Run 1**	$I1_j$	$I2_j$	$I3_j$	$W1_j$	$W2_j$	$W3_j$	$N1_j$	$N2_j$	$N3_j$	$S1_j$	$S2_j$	$S3_j$	$S4_j$	$S5_j$	$S6_j$
$I1_i$	**0.6850<br>(0.0180)**	0.0454<br>(0.0389)	0.0179<br>(0.0171)	0.0183<br>(0.0185)	0.0177<br>(0.0168)	0.0330<br>(0.0280)	0.0179<br>(0.0169)	0.0405<br>(0.0306)	0.0175<br>(0.0164)	0.0179<br>(0.0168)	0.0186<br>(0.0183)	0.0181<br>(0.0172)	0.0176<br>(0.0169)	0.0173<br>(0.0164)	0.0174<br>(0.0164)
$I2_i$	0.0136<br>(0.0132)	**0.7980<br>(0.0339)**	0.0136<br>(0.0129)	0.0134<br>(0.0131)	0.0136<br>(0.0131)	0.0168<br>(0.0153)	0.0136<br>(0.0130)	0.0160<br>(0.0155)	0.0135<br>(0.0130)	0.0173<br>(0.0159)	0.0168<br>(0.0159)	0.0135<br>(0.0130)	0.0135<br>(0.0131)	0.0135<br>(0.0130)	0.0133<br>(0.0126)
$I3_i$	0.0144<br>(0.0139)	0.1189<br>(0.0356)	**0.6806<br>(0.0134)**	0.0140<br>(0.0134)	0.0211<br>(0.0185)	0.0140<br>(0.0133)	0.0140<br>(0.0133)	0.0232<br>(0.0199)	0.0144<br>(0.0137)	0.0139<br>(0.0131)	0.0146<br>(0.0142)	0.0153<br>(0.0145)	0.0138<br>(0.0132)	0.0140<br>(0.0132)	0.0138<br>(0.0132)
$W1_i$	0.0175<br>(0.0169)	0.0203<br>(0.0192)	0.0174<br>(0.0164)	**0.6842<br>(0.0165)**	0.0179<br>(0.0171)	0.0189<br>(0.0178)	0.0174<br>(0.0165)	0.0793<br>(0.0330)	0.0177<br>(0.0167)	0.0175<br>(0.0167)	0.0177<br>(0.0169)	0.0174<br>(0.0168)	0.0179<br>(0.0170)	0.0212<br>(0.0195)	0.0176<br>(0.0167)
$W2_i$	0.0080<br>(0.0079)	0.0101<br>(0.0094)	0.0079<br>(0.0077)	0.0081<br>(0.0080)	**0.8792<br>(0.0257)**	0.0080<br>(0.0079)	0.0080<br>(0.0079)	0.0105<br>(0.0100)	0.0080<br>(0.0079)	0.0087<br>(0.0085)	0.0086<br>(0.0083)	0.0081<br>(0.0078)	0.0081<br>(0.0078)	0.0105<br>(0.0099)	0.0082<br>(0.0081)
$W3_i$	0.0088<br>(0.0087)	0.0088<br>(0.0086)	0.0088<br>(0.0087)	0.0087<br>(0.0085)	0.0089<br>(0.0087)	**0.8694<br>(0.0270)**	0.0089<br>(0.0086)	0.0098<br>(0.0095)	0.0087<br>(0.0085)	0.0088<br>(0.0086)	0.0087<br>(0.0087)	0.0092<br>(0.0089)	0.0088<br>(0.0085)	0.0149<br>(0.0124)	0.0088<br>(0.0085)
$N1_i$	0.0185<br>(0.0175)	0.0181<br>(0.0175)	0.0187<br>(0.0176)	0.0186<br>(0.0173)	0.0191<br>(0.0177)	0.0193<br>(0.0181)	**0.6849<br>(0.0171)**	0.0711<br>(0.0320)	0.0187<br>(0.0177)	0.0187<br>(0.0179)	0.0185<br>(0.0178)	0.0203<br>(0.0194)	0.0185<br>(0.0175)	0.0186<br>(0.0178)	0.0183<br>(0.0175)
$N2_i$	0.0119<br>(0.0114)	0.0119<br>(0.0113)	0.0122<br>(0.0117)	0.0120<br>(0.0116)	0.0138<br>(0.0134)	0.0123<br>(0.0117)	0.0121<br>(0.0117)	**0.8201<br>(0.0329)**	0.0119<br>(0.0115)	0.0129<br>(0.0126)	0.0138<br>(0.0131)	0.0143<br>(0.0134)	0.0119<br>(0.0116)	0.0156<br>(0.0144)	0.0134<br>(0.0131)
$N3_i$	0.0063<br>(0.0063)	0.0065<br>(0.0064)	0.0064<br>(0.0064)	0.0064<br>(0.0063)	0.0064<br>(0.0064)	0.0065<br>(0.0063)	0.0065<br>(0.0065)	0.0066<br>(0.0064)	**0.9055<br>(0.0216)**	0.0064<br>(0.0061)	0.0086<br>(0.0086)	0.0086<br>(0.0078)	0.0064<br>(0.0063)	0.0064<br>(0.0063)	0.0065<br>(0.0064)
$S1_i$	0.0132<br>(0.0127)	0.0134<br>(0.0131)	0.0133<br>(0.0127)	0.0136<br>(0.0134)	0.0134<br>(0.0127)	0.0135<br>(0.0126)	0.0133<br>(0.0129)	0.0136<br>(0.0131)	0.0135<br>(0.0127)	**0.7483<br>(0.0703)**	0.0768<br>(0.0705)	0.0136<br>(0.0130)	0.0133<br>(0.0129)	0.0135<br>(0.0129)	0.0138<br>(0.0133)
$S2_i$	0.0088<br>(0.0085)	0.0087<br>(0.0084)	0.0088<br>(0.0086)	0.0088<br>(0.0086)	0.0088<br>(0.0085)	0.0088<br>(0.0086)	0.0088<br>(0.0084)	0.0088<br>(0.0085)	0.0973<br>(0.0967)	0.0094<br>(0.0090)	**0.7873<br>(0.0966)**	0.0095<br>(0.0092)	0.0088<br>(0.0086)	0.0087<br>(0.0085)	0.0086<br>(0.0086)
$S3_i$	0.0081<br>(0.0080)	0.0081<br>(0.0080)	0.0081<br>(0.0079)	0.0081<br>(0.0080)	0.0081<br>(0.0079)	0.0087<br>(0.0083)	0.0080<br>(0.0079)	0.0088<br>(0.0085)	0.0098<br>(0.0094)	0.0082<br>(0.0078)	0.0088<br>(0.0086)	**0.8814<br>(0.0253)**	0.0081<br>(0.0080)	0.0085<br>(0.0083)	0.0092<br>(0.0089)
$S4_i$	0.0122<br>(0.0117)	0.0124<br>(0.0120)	0.0125<br>(0.0120)	0.0125<br>(0.0123)	0.0124<br>(0.0119)	0.0124<br>(0.0120)	0.0122<br>(0.0118)	0.0131<br>(0.0126)	0.0136<br>(0.0133)	0.0125<br>(0.0119)	0.0125<br>(0.0120)	0.0143<br>(0.0136)	**0.6792<br>(0.0121)**	0.1557<br>(0.0323)	0.0125<br>(0.0120)
$S5_i$	0.0127<br>(0.0121)	0.0125<br>(0.0122)	0.0129<br>(0.0124)	0.0129<br>(0.0125)	0.0127<br>(0.0121)	0.0150<br>(0.0144)	0.0130<br>(0.0123)	0.0131<br>(0.0126)	0.0129<br>(0.0123)	0.0127<br>(0.0124)	0.0129<br>(0.0124)	0.0129<br>(0.0124)	0.0127<br>(0.0122)	**0.8180<br>(0.0322)**	0.0130<br>(0.0126)
$S6_i$	0.0060<br>(0.0059)	0.0059<br>(0.0058)	0.0059<br>(0.0058)	0.0060<br>(0.0058)	0.0059<br>(0.0059)	0.0061<br>(0.0060)	0.0060<br>(0.0058)	0.0060<br>(0.0060)	0.0059<br>(0.0058)	0.0059<br>(0.0058)	0.0059<br>(0.0058)	0.0061<br>(0.0060)	0.0060<br>(0.0059)	0.0059<br>(0.0058)	**0.9164<br>(0.0193)**

**Run 2**	$I1_j$	$I2_j$	$I3_j$	$W1_j$	$W2_j$	$W3_j$	$N1_j$	$N2_j$	$N3_j$	$S1_j$	$S2_j$	$S3_j$	$S4_j$	$S5_j$	$S6_j$
$I1_i$	**0.6848<br>(0.0172)**	0.0418<br>(0.0393)	0.0399<br>(0.0400)	0.0175<br>(0.0163)	0.0175<br>(0.0165)	0.0262<br>(0.0246)	0.0178<br>(0.0169)	0.0306<br>(0.0289)	0.0176<br>(0.0164)	0.0192<br>(0.0188)	0.0174<br>(0.0166)	0.0176<br>(0.0167)	0.0174<br>(0.0164)	0.0174<br>(0.0165)	0.0173<br>(0.0166)
$I2_i$	0.0135<br>(0.0132)	**0.7976<br>(0.0340)**	0.0145<br>(0.0139)	0.0138<br>(0.0135)	0.0138<br>(0.0131)	0.0166<br>(0.0153)	0.0132<br>(0.0126)	0.0154<br>(0.0146)	0.0132<br>(0.0126)	0.0203<br>(0.0179)	0.0142<br>(0.0134)	0.0134<br>(0.0129)	0.0135<br>(0.0131)	0.0134<br>(0.0127)	0.0136<br>(0.0129)
$I3_i$	0.0138<br>(0.0132)	**0.1111<br>(0.0383)**	**0.6858<br>(0.0186)**	0.0139<br>(0.0133)	0.0235<br>(0.0197)	0.0138<br>(0.0131)	0.0140<br>(0.0138)	0.0236<br>(0.0202)	0.0143<br>(0.0137)	0.0143<br>(0.0137)	0.0148<br>(0.0140)	0.0151<br>(0.0144)	0.0141<br>(0.0136)	0.0140<br>(0.0134)	0.0140<br>(0.0134)
$W1_i$	0.0175<br>(0.0167)	0.0204<br>(0.0189)	0.0179<br>(0.0172)	**0.6839<br>(0.0163)**	0.0173<br>(0.0167)	0.0192<br>(0.0180)	0.0176<br>(0.0168)	**0.0805<br>(0.0335)**	0.0175<br>(0.0170)	0.0170<br>(0.0163)	0.0182<br>(0.0175)	0.0175<br>(0.0166)	0.0177<br>(0.0169)	0.0201<br>(0.0185)	0.0176<br>(0.0167)
$W2_i$	0.0080<br>(0.0077)	0.0097<br>(0.0093)	0.0091<br>(0.0087)	0.0080<br>(0.0079)	**0.8789<br>(0.0260)**	0.0082<br>(0.0081)	0.0080<br>(0.0079)	0.0101<br>(0.0097)	0.0081<br>(0.0080)	0.0093<br>(0.0091)	0.0082<br>(0.0080)	0.0081<br>(0.0079)	0.0080<br>(0.0078)	0.0104<br>(0.0096)	0.0079<br>(0.0078)
$W3_i$	0.0087<br>(0.0086)	0.0089<br>(0.0086)	0.0087<br>(0.0085)	0.0087<br>(0.0084)	0.0089<br>(0.0086)	**0.8697<br>(0.0270)**	0.0088<br>(0.0085)	0.0099<br>(0.0096)	0.0088<br>(0.0085)	0.0086<br>(0.0085)	0.0088<br>(0.0085)	0.0091<br>(0.0088)	0.0087<br>(0.0085)	0.0149<br>(0.0124)	0.0088<br>(0.0085)
$N1_i$	0.0186<br>(0.0174)	0.0186<br>(0.0175)	0.0187<br>(0.0176)	0.0181<br>(0.0174)	0.0186<br>(0.0176)	0.0189<br>(0.0181)	**0.6854<br>(0.0178)**	**0.0713<br>(0.0319)**	0.0182<br>(0.0171)	0.0188<br>(0.0176)	0.0184<br>(0.0175)	0.0202<br>(0.0190)	0.0185<br>(0.0177)	0.0188<br>(0.0180)	0.0188<br>(0.0178)
$N2_i$	0.0118<br>(0.0114)	0.0120<br>(0.0118)	0.0119<br>(0.0116)	0.0118<br>(0.0113)	0.0138<br>(0.0132)	0.0124<br>(0.0119)	0.0116<br>(0.0114)	**0.8204<br>(0.0323)**	0.0121<br>(0.0115)	0.0135<br>(0.0128)	0.0137<br>(0.0131)	0.0143<br>(0.0137)	0.0119<br>(0.0115)	0.0154<br>(0.0144)	0.0134<br>(0.0128)
$N3_i$	0.0065<br>(0.0064)	0.0064<br>(0.0062)	0.0065<br>(0.0065)	0.0064<br>(0.0063)	0.0066<br>(0.0063)	0.0064<br>(0.0063)	0.0064<br>(0.0063)	0.0066<br>(0.0064)	**0.9034<br>(0.0216)**	0.0064<br>(0.0064)	0.0102<br>(0.0097)	0.0090<br>(0.0080)	0.0065<br>(0.0064)	0.0064<br>(0.0063)	0.0064<br>(0.0062)
$S1_i$	0.0134<br>(0.0128)	0.0133<br>(0.0126)	0.0132<br>(0.0126)	0.0132<br>(0.0127)	0.0133<br>(0.0127)	0.0135<br>(0.0132)	0.0135<br>(0.0130)	0.0138<br>(0.0130)	0.0132<br>(0.0127)	**0.8033<br>(0.0452)**	0.0219<br>(0.0358)	0.0135<br>(0.0130)	0.0135<br>(0.0129)	0.0133<br>(0.0129)	0.0140<br>(0.0133)
$S2_i$	0.0087<br>(0.0085)	0.0090<br>(0.0087)	0.0088<br>(0.0086)	0.0088<br>(0.0087)	0.0088<br>(0.0084)	0.0089<br>(0.0087)	0.0086<br>(0.0084)	0.0088<br>(0.0086)	0.0244<br>(0.0399)	0.0097<br>(0.0093)	**0.8593<br>(0.0457)**	0.0096<br>(0.0093)	0.0088<br>(0.0085)	0.0088<br>(0.0087)	0.0090<br>(0.0087)
$S3_i$	0.0082<br>(0.0079)	0.0081<br>(0.0079)	0.0082<br>(0.0080)	0.0083<br>(0.0080)	0.0081<br>(0.0079)	0.0088<br>(0.0084)	0.0082<br>(0.0079)	0.0089<br>(0.0086)	0.0097<br>(0.0092)	0.0082<br>(0.0081)	0.0096<br>(0.0092)	**0.8802<br>(0.0255)**	0.0081<br>(0.0079)	0.0086<br>(0.0083)	0.0090<br>(0.0086)
$S4_i$	0.0123<br>(0.0119)	0.0123<br>(0.0119)	0.0122<br>(0.0118)	0.0123<br>(0.0117)	0.0123<br>(0.0119)	0.0122<br>(0.0116)	0.0123<br>(0.0116)	0.0133<br>(0.0126)	0.0135<br>(0.0131)	0.0123<br>(0.0118)	0.0129<br>(0.0125)	0.0144<br>(0.0137)	**0.6790<br>(0.0117)**	**0.1564<br>(0.0318)**	0.0124<br>(0.0120)
$S5_i$	0.0130<br>(0.0125)	0.0128<br>(0.0125)	0.0128<br>(0.0121)	0.0128<br>(0.0124)	0.0130<br>(0.0125)	0.0149<br>(0.0141)	0.0126<br>(0.0122)	0.0129<br>(0.0123)	0.0128<br>(0.0122)	0.0129<br>(0.0125)	0.0132<br>(0.0128)	0.0128<br>(0.0124)	0.0126<br>(0.0120)	**0.8182<br>(0.0322)**	0.0128<br>(0.0123)
$S6_i$	0.0059<br>(0.0059)	0.0060<br>(0.0058)	0.0060<br>(0.0059)	0.0059<br>(0.0058)	0.0059<br>(0.0058)	0.0061<br>(0.0060)	0.0060<br>(0.0060)	0.0061<br>(0.0060)	0.0061<br>(0.0060)	0.0060<br>(0.0058)	0.0060<br>(0.0058)	0.0062<br>(0.0062)	0.0060<br>(0.0057)	0.0061<br>(0.0061)	**0.9157<br>(0.0194)**

**Run 3**	$I1_j$	$I2_j$	$I3_j$	$W1_j$	$W2_j$	$W3_j$	$N1_j$	$N2_j$	$N3_j$	$S1_j$	$S2_j$	$S3_j$	$S4_j$	$S5_j$	$S6_j$
$I1_i$	**0.6852 (0.0185)**	0.0557 (0.0411)	0.0219 (0.0246)	0.0177 (0.0170)	0.0175 (0.0167)	0.0259 (0.0244)	0.0178 (0.0169)	0.0338 (0.0299)	0.0174 (0.0164)	0.0187 (0.0180)	0.0180 (0.0177)	0.0175 (0.0167)	0.0177 (0.0167)	0.0178 (0.0171)	0.0175 (0.0164)
$I2_i$	0.0134 (0.0130)	**0.7989 (0.0338)**	0.0139 (0.0132)	0.0135 (0.0128)	0.0136 (0.0131)	0.0171 (0.0158)	0.0134 (0.0129)	0.0156 (0.0150)	0.0133 (0.0130)	0.0179 (0.0165)	0.0156 (0.0149)	0.0133 (0.0127)	0.0135 (0.0128)	0.0135 (0.0130)	0.0135 (0.0131)
$I3_i$	0.0143 (0.0136)	**0.1194 (0.0364)**	**0.6813 (0.0143)**	0.0139 (0.0133)	0.0211 (0.0188)	0.0139 (0.0132)	0.0137 (0.0133)	0.0222 (0.0196)	0.0143 (0.0138)	0.0142 (0.0137)	0.0146 (0.0141)	0.0150 (0.0144)	0.0139 (0.0133)	0.0141 (0.0135)	0.0140 (0.0135)
$W1_i$	0.0176 (0.0167)	0.0207 (0.0194)	0.0174 (0.0165)	**0.6845 (0.0168)**	0.0176 (0.0170)	0.0185 (0.0175)	0.0175 (0.0168)	**0.0801 (0.0331)**	0.0177 (0.0167)	0.0176 (0.0168)	0.0177 (0.0169)	0.0175 (0.0165)	0.0174 (0.0162)	0.0209 (0.0192)	0.0172 (0.0164)
$W2_i$	0.0081 (0.0079)	0.0099 (0.0093)	0.0081 (0.0079)	0.0082 (0.0078)	**0.8791 (0.0253)**	0.0081 (0.0079)	0.0080 (0.0079)	0.0104 (0.0099)	0.0080 (0.0079)	0.0088 (0.0085)	0.0083 (0.0081)	0.0083 (0.0081)	0.0080 (0.0078)	0.0104 (0.0097)	0.0082 (0.0079)
$W3_i$	0.0088 (0.0086)	0.0088 (0.0085)	0.0087 (0.0086)	0.0088 (0.0087)	0.0087 (0.0086)	**0.8698 (0.0270)**	0.0089 (0.0084)	0.0097 (0.0092)	0.0088 (0.0086)	0.0087 (0.0084)	0.0088 (0.0086)	0.0091 (0.0086)	0.0087 (0.0083)	0.0148 (0.0122)	0.0088 (0.0086)
$N1_i$	0.0185 (0.0178)	0.0186 (0.0178)	0.0183 (0.0172)	0.0184 (0.0175)	0.0188 (0.0178)	0.0194 (0.0185)	**0.6848 (0.0173)**	**0.0720 (0.0326)**	0.0183 (0.0172)	0.0183 (0.0172)	0.0188 (0.0179)	0.0202 (0.0187)	0.0185 (0.0177)	0.0187 (0.0177)	0.0183 (0.0175)
$N2_i$	0.0120 (0.0115)	0.0120 (0.0115)	0.0117 (0.0114)	0.0119 (0.0117)	0.0138 (0.0134)	0.0123 (0.0118)	0.0119 (0.0116)	**0.8204 (0.0323)**	0.0121 (0.0117)	0.0132 (0.0128)	0.0139 (0.0133)	0.0141 (0.0132)	0.0119 (0.0116)	0.0154 (0.0145)	0.0133 (0.0128)
$N3_i$	0.0065 (0.0065)	0.0064 (0.0063)	0.0064 (0.0063)	0.0065 (0.0064)	0.0065 (0.0064)	0.0064 (0.0062)	0.0065 (0.0063)	0.0065 (0.0065)	**0.9046 (0.0216)**	0.0065 (0.0063)	0.0091 (0.0089)	0.0086 (0.0079)	0.0064 (0.0063)	0.0065 (0.0063)	0.0066 (0.0064)
$S1_i$	0.0132 (0.0126)	0.0133 (0.0127)	0.0133 (0.0127)	0.0132 (0.0127)	0.0136 (0.0129)	0.0134 (0.0129)	0.0134 (0.0126)	0.0135 (0.0132)	0.0133 (0.0127)	**0.7636 (0.0693)**	0.0623 (0.0674)	0.0136 (0.0130)	0.0132 (0.0126)	0.0133 (0.0130)	0.0139 (0.0133)
$S2_i$	0.0087 (0.0085)	0.0088 (0.0086)	0.0088 (0.0087)	0.0087 (0.0085)	0.0086 (0.0084)	0.0090 (0.0087)	0.0087 (0.0084)	0.0089 (0.0087)	0.0836 (0.0938)	0.0092 (0.0089)	**0.8010 (0.0933)**	0.0092 (0.0089)	0.0087 (0.0084)	0.0089 (0.0090)	0.0090 (0.0087)
$S3_i$	0.0081 (0.0078)	0.0083 (0.0081)	0.0081 (0.0079)	0.0083 (0.0080)	0.0082 (0.0079)	0.0088 (0.0086)	0.0083 (0.0081)	0.0088 (0.0085)	0.0098 (0.0095)	0.0080 (0.0080)	0.0090 (0.0089)	**0.8810 (0.0253)**	0.0081 (0.0079)	0.0084 (0.0083)	0.0089 (0.0086)
$S4_i$	0.0123 (0.0116)	0.0125 (0.0120)	0.0123 (0.0118)	0.0125 (0.0120)	0.0124 (0.0118)	0.0124 (0.0117)	0.0121 (0.0117)	0.0128 (0.0123)	0.0132 (0.0125)	0.0125 (0.0121)	0.0129 (0.0125)	0.0145 (0.0136)	**0.6792 (0.0121)**	**0.1560 (0.0322)**	0.0124 (0.0122)
$S5_i$	0.0129 (0.0126)	0.0127 (0.0123)	0.0128 (0.0123)	0.0129 (0.0124)	0.0129 (0.0124)	0.0150 (0.0142)	0.0129 (0.0124)	0.0130 (0.0127)	0.0129 (0.0123)	0.0127 (0.0122)	0.0130 (0.0124)	0.0130 (0.0126)	0.0128 (0.0121)	**0.8176 (0.0324)**	0.0129 (0.0124)
$S6_i$	0.0059 (0.0058)	0.0060 (0.0059)	0.0059 (0.0058)	0.0061 (0.0060)	0.0059 (0.0060)	0.0060 (0.0058)	0.0060 (0.0059)	0.0060 (0.0060)	0.0059 (0.0058)	0.0058 (0.0058)	0.0061 (0.0059)	0.0060 (0.0059)	0.0059 (0.0059)	0.0059 (0.0057)	**0.9165 (0.0192)**
