# Supplementary material for: Landscape and climatic features drive genetic differentiation processes in a South American coastal plant
Source: BMC Ecol Evol. 2021 Oct 26;21:196. doi: 10.1186/s12862-021-01916-4 (PMC8547116; doi:10.1186/s12862-021-01916-4)
Supplement: Supplementary file 2 — Additional file 2: Migrate-n detailed output for the best-supported model showing parameter estimation values and convergence statistics. [file 12862_2021_1916_MOESM2_ESM.pdf]

MIGRATION RATE AND POPULATION SIZE ESTIMATION
using the coalescent and maximum likelihood or Bayesian inference
Migrate-n version 3.6.11 [June-18-15]
Compiled for a PARALLEL COMPUTER ARCHITECTURE
One master and 10 compute nodes are available.
Program started at Sat May 14 12:22:20 2016
Program finished at Mon May 16 20:06:30 2016

Options

Datatype: Microsatellite data [Singlestep model]
Missing data: not included

Inheritance scalers in use for Thetas:
All loci use an inheritance scaler of 1.0
[The locus with a scaler of 1.0 used as reference]
Random number seed: (with internal timer) 398713161
Start parameters:

Theta values were generated from the FST-calculation

M values were generated from the FST-calculation

Connection type matrix:
where m = average (average over a group of Thetas or M,
s = symmetric M, S = symmetric 4Nm, 0 = zero, and not estimated,
* = free to vary, Thetas are on diagonal

Population	1	2	3	4
1 pop_01	*	*	0	0
2 pop_02	0	*	*	*
3 pop_03	0	0	*	0
4 pop_04	0	0	0	*

Order of parameters:
1 Θ_1 <displayed>

2	Θ_2	<displayed>
3	Θ_3	<displayed>
4	Θ_4	<displayed>
5	$M_{2->1}$	<displayed>
9	$M_{3->2}$	<displayed>
10	$M_{4->2}$	<displayed>

Mutation rate among loci: Varying ([crudely] estimated from data)

Rates per locus: 1.58940, 0.52980, 1.25828, 0.59603, 0.66225,
1.92053, 0.52980, 0.72848, 0.66225, 1.52318

Analysis strategy: Bayesian inference

Proposal distributions for parameter

Parameter	Proposal
Theta	Slice sampling
M	Slice sampling

Prior distribution for parameter

Parameter	Prior	Minimum	Mean*	Maximum	Delta	Bins
Theta	Uniform	0.000000	10.000000	20.000000	0.500000	1500
M	Uniform	0.000000	10.000000	20.000000	0.500000	1500

Markov chain settings: Long chain

Number of chains	1
Recorded steps [a]	50000
Increment (record every x step [b]	100
Number of concurrent chains (replicates) [c]	1
Visited (sampled) parameter values [a*b*c]	5000000
Number of discard trees per chain (burn-in)	30000

Multiple Markov chains:

Static heating scheme 4 chains with temperatures

1000000.00 3.00 1.50 1.00

Swapping interval is 10

Print options:

Data file:	infile
Output file:	Che_step_stone_c2i
Posterior distribution raw histogram file:	bayesfile
Print data:	Yes
Print genealogies [only some for some data type]:	None

Bayesian Analysis: Posterior distribution table

Locus	Parameter	2.5%	25.0%	Mode	75.0%	97.5%	Median	Mean
1	Θ_1	0.00000	2.60000	0.00000	0.00000	16.54667	0.00000	0.00000
1	Θ_2	0.00000	16.29333	0.00000	0.00000	19.94667	0.00000	0.00000
1	Θ_3	0.00000	1.24000	0.00000	0.00000	5.24000	0.00000	0.00000
1	Θ_4	0.00000	0.00000	0.00000	0.00000	0.33333	0.00000	0.00000
1	$M_{2->1}$	0.000	0.173	0.000	0.000	3.653	0.000	0.000
1	$M_{3->2}$	0.000	0.000	0.000	0.000	0.440	0.000	0.000
1	$M_{4->2}$	0.000	0.000	0.000	0.000	0.280	0.000	0.000
2	Θ_1	0.00000	1.16000	0.00000	0.00000	17.84000	0.00000	0.00000
2	Θ_2	0.00000	0.72000	0.00000	0.00000	15.28000	0.00000	0.00000
2	Θ_3	0.00000	0.06667	0.00000	0.00000	0.69333	0.00000	0.00000
2	Θ_4	0.00000	5.72000	0.00000	0.00000	13.96000	0.00000	0.00000
2	$M_{2->1}$	0.000	13.720	0.000	0.000	20.000	0.000	0.000
2	$M_{3->2}$	0.000	0.000	0.000	0.000	1.413	0.000	0.000
2	$M_{4->2}$	3.120	0.000	0.000	0.000	17.253	0.000	0.000
3	Θ_1	0.00000	2.46667	0.00000	0.00000	13.57333	0.00000	0.00000
3	Θ_2	0.00000	0.13333	0.00000	0.00000	0.77333	0.00000	0.00000
3	Θ_3	0.00000	0.73333	0.00000	0.00000	1.46667	0.00000	0.00000
3	Θ_4	0.00000	2.94667	0.00000	0.00000	12.49333	0.00000	0.00000
3	$M_{2->1}$	0.000	0.053	0.000	0.000	4.120	0.000	0.000
3	$M_{3->2}$	4.520	0.000	0.000	0.000	19.867	0.000	0.000
3	$M_{4->2}$	6.293	0.000	0.000	0.000	20.000	0.000	0.000
4	Θ_1	0.00000	1.94667	0.00000	0.00000	16.72000	0.00000	0.00000
4	Θ_2	0.00000	0.89333	0.00000	0.00000	15.40000	0.00000	0.00000
4	Θ_3	0.00000	0.69333	0.00000	0.00000	2.49333	0.00000	0.00000
4	Θ_4	0.00000	5.81333	0.00000	0.00000	12.10667	0.00000	0.00000
4	$M_{2->1}$	0.000	0.067	0.000	0.000	1.800	0.000	0.000
4	$M_{3->2}$	0.000	0.000	0.000	0.000	2.933	0.000	0.000
4	$M_{4->2}$	9.093	0.000	0.000	0.000	20.000	0.000	0.000
5	Θ_1	0.00000	0.38667	0.00000	0.00000	17.77333	0.00000	0.00000
5	Θ_2	0.00000	0.28000	0.00000	0.00000	3.29333	0.00000	0.00000
5	Θ_3	0.00000	2.26667	0.00000	0.00000	6.12000	0.00000	0.00000
5	Θ_4	0.00000	0.54667	0.00000	0.00000	1.34667	0.00000	0.00000

Locus	Parameter	2.5%	25.0%	Mode	75.0%	97.5%	Median	Mean
5	$M_{2->1}$	0.000	9.613	0.000	0.000	19.960	0.000	0.000
5	$M_{3->2}$	1.120	0.000	0.000	0.000	16.080	0.000	0.000
5	$M_{4->2}$	0.000	0.000	0.000	0.000	5.507	0.000	0.000
6	Θ_1	0.00000	9.42667	0.00000	0.00000	20.00000	0.00000	0.00000
6	Θ_2	0.00000	5.82667	0.00000	0.00000	10.49333	0.00000	0.00000
6	Θ_3	0.00000	0.17333	0.00000	0.00000	0.72000	0.00000	0.00000
6	Θ_4	0.00000	5.10667	0.00000	0.00000	9.05333	0.00000	0.00000
6	$M_{2->1}$	0.000	0.440	0.000	0.000	4.293	0.000	0.000
6	$M_{3->2}$	0.000	0.000	0.000	0.000	0.320	0.000	0.000
6	$M_{4->2}$	0.000	0.000	0.000	0.000	1.267	0.000	0.000
7	Θ_1	0.00000	5.62667	0.00000	0.00000	19.69333	0.00000	0.00000
7	Θ_2	0.00000	0.06667	0.00000	0.00000	8.50667	0.00000	0.00000
7	Θ_3	0.00000	0.61333	0.00000	0.00000	2.49333	0.00000	0.00000
7	Θ_4	0.00000	1.12000	0.00000	0.00000	6.50667	0.00000	0.00000
7	$M_{2->1}$	0.000	0.867	0.000	0.000	6.507	0.000	0.000
7	$M_{3->2}$	0.000	0.000	0.000	0.000	6.373	0.000	0.000
7	$M_{4->2}$	8.187	0.000	0.000	0.000	20.000	0.000	0.000
8	Θ_1	0.00000	0.10667	0.00000	0.00000	7.50667	0.00000	0.00000
8	Θ_2	0.00000	1.85333	0.00000	0.00000	17.28000	0.00000	0.00000
8	Θ_3	0.00000	2.16000	0.00000	0.00000	7.21333	0.00000	0.00000
8	Θ_4	0.00000	0.33333	0.00000	0.00000	1.04000	0.00000	0.00000
8	$M_{2->1}$	0.000	0.333	0.000	0.000	15.960	0.000	0.000
8	$M_{3->2}$	0.107	0.000	0.000	0.000	4.027	0.000	0.000
8	$M_{4->2}$	0.000	0.000	0.000	0.000	0.373	0.000	0.000
9	Θ_1	0.00000	3.17333	0.00000	0.00000	16.30667	0.00000	0.00000
9	Θ_2	0.00000	4.12000	0.00000	0.00000	17.38667	0.00000	0.00000
9	Θ_3	0.00000	1.69333	0.00000	0.00000	5.93333	0.00000	0.00000
9	Θ_4	0.00000	0.00000	0.00000	0.00000	0.41333	0.00000	0.00000
9	$M_{2->1}$	0.000	0.147	0.000	0.000	2.547	0.000	0.000
9	$M_{3->2}$	0.000	0.000	0.000	0.000	1.533	0.000	0.000
9	$M_{4->2}$	0.000	0.000	0.000	0.000	0.307	0.000	0.000
10	Θ_1	0.00000	2.33333	0.00000	0.00000	18.64000	0.00000	0.00000
10	Θ_2	0.00000	4.12000	0.00000	0.00000	18.88000	0.00000	0.00000
10	Θ_3	0.00000	5.73333	0.00000	0.00000	12.64000	0.00000	0.00000
10	Θ_4	0.00000	0.00000	0.00000	0.00000	0.38667	0.00000	0.00000
10	$M_{2->1}$	0.000	0.053	0.000	0.000	14.427	0.000	0.000

Locus	Parameter	2.5%	25.0%	Mode	75.0%	97.5%	Median	Mean
10	$M_{3->2}$	0.093	0.000	0.000	0.000	2.173	0.000	0.000
10	$M_{4->2}$	0.000	0.000	0.000	0.000	0.320	0.000	0.000
All	Θ_1	2.54667	3.38667	3.90000	4.49333	6.28000	4.11333	4.25062
All	Θ_2	0.12000	0.37333	0.54000	0.70667	0.96000	0.55333	0.55799
All	Θ_3	0.05333	0.32000	0.50000	0.65333	0.94667	0.51333	0.52469
All	Θ_4	0.00000	0.06667	0.20667	0.33333	0.54667	0.27333	0.22232
All	$M_{2->1}$	0.000	0.080	0.287	0.480	1.160	0.407	0.446
All	$M_{3->2}$	0.000	0.120	0.260	0.400	0.600	0.300	0.263
All	$M_{4->2}$	0.000	0.000	0.073	0.187	0.413	0.193	0.078

Citation suggestions:

Beerli P., 2006. Comparison of Bayesian and maximum-likelihood inference of population genetic parameters. Bioinformatics 22:341-345

Beerli P., 2007. Estimation of the population scaled mutation rate from microsatellite data, Genetics, 177:1967-1968.

Beerli P., 2009. How to use MIGRATE or why are Markov chain Monte Carlo programs difficult to use? In Population Genetics for Animal Conservation, G. Bertorelle, M. W. Bruford, H. C. Hauffe, A. Rizzoli, and C. Vernesi, eds., vol. 17 of Conservation Biology, Cambridge University Press, Cambridge UK, pp. 42-79.

Bayesian Analysis: Posterior distribution over all loci

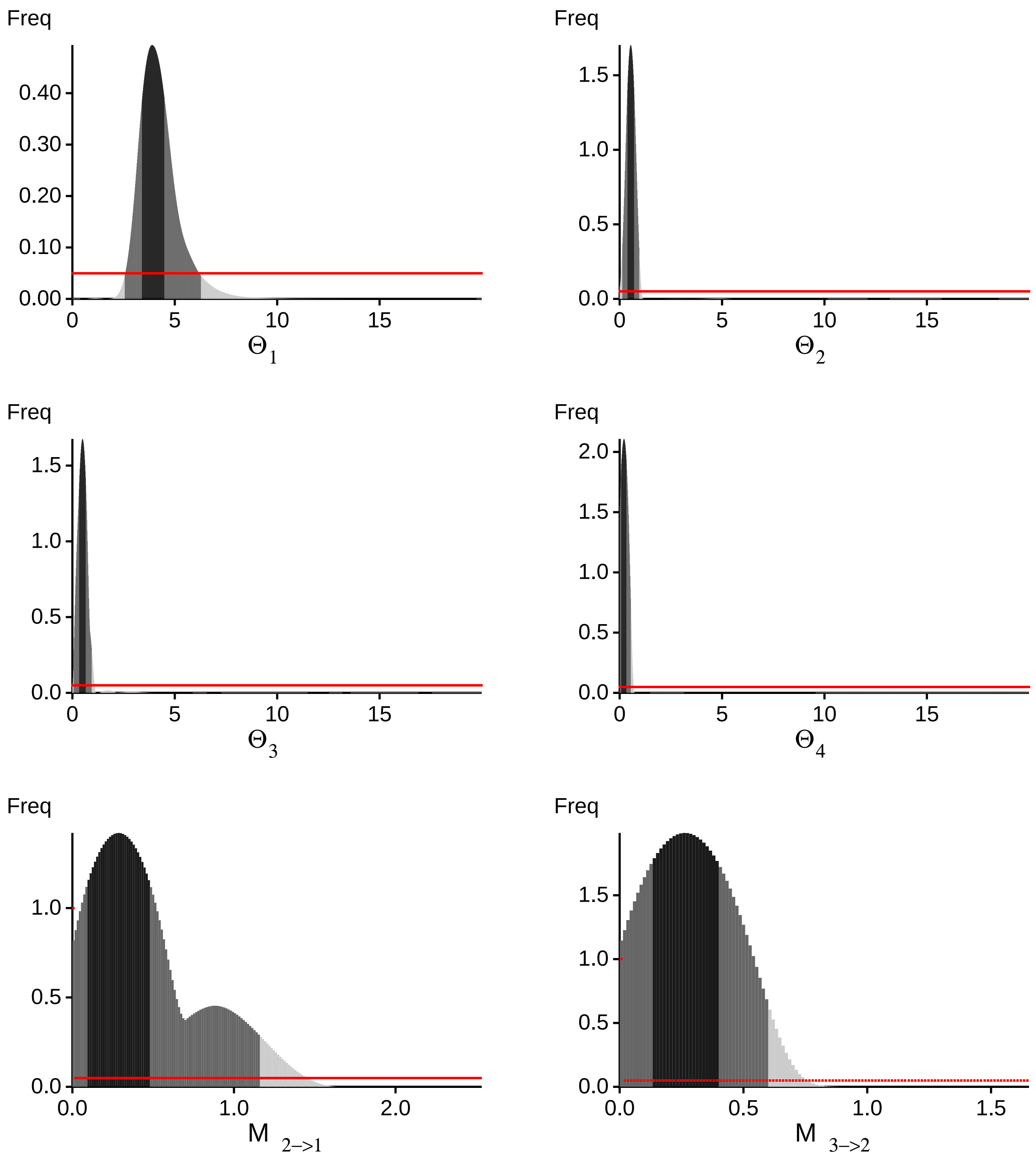

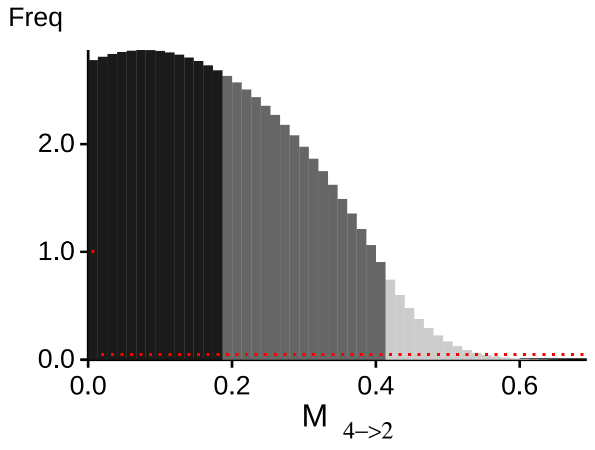
Freq
2.0
1.0
0.0
0.0
0.2
0.4
0.6
M 4->2

Log-Probability of the data given the model (marginal likelihood)

Use this value for Bayes factor calculations:
BF = Exp[ln(Prob(D | thisModel) - ln(Prob(D | otherModel)
or as LBF = 2 (ln(Prob(D | thisModel) - ln(Prob(D | otherModel))
shows the support for thisModel]

Locus	Raw thermodynamic score(1a)	Bezier approximation score(1b)	Harmonic mean(2)
1	-2109.02	-932.23	-394.72
2	-830.01	-469.40	-171.47
3	-1262.87	-780.05	-275.27
4	-814.85	-593.87	-181.69
5	-533.79	-272.85	-108.87
6	-2487.64	-898.46	-297.62
7	-411.70	-262.39	-100.48
8	-671.50	-523.80	-203.33
9	-1031.65	-557.58	-214.38
10	-1506.13	-673.39	-302.58
All	-11595.81	-5900.67	-2187.06

(1a, 1b and 2) are approximations to the marginal likelihood, make sure that the program run long enough!
(1a, 1b) and (2) should give similar results, in principle.
But (2) is overestimating the likelihood, it is presented for historical reasons and should not be used
(1a, 1b) needs heating with chains that span a temperature range of 1.0 to at least 100,000.
(1b) is using a Bezier-curve to get better approximations for runs with low number of heated chains
[Scaling factor = 63.345791
Citation suggestions:

Beerli P. and M. Palczewski, 2010. Unified framework to evaluate panmixia and migration direction among multiple sampling locations, Genetics, 185: 313-326.

Acceptance ratios for all parameters and the genealogies

Parameter	Accepted changes	Ratio
Θ_1	5354193/5354193	1.00000
Θ_2	5362339/5362339	1.00000
Θ_3	5356678/5356678	1.00000
Θ_4	5354879/5354879	1.00000
$M_{2->1}$	5355619/5355619	1.00000
$M_{3->2}$	5356616/5356616	1.00000
$M_{4->2}$	5357676/5357676	1.00000
Genealogies	4571818/12502000	0.36569

MCMC-Autocorrelation and Effective MCMC Sample Size

Parameter	Autocorrelation	Effective Sampe Size
Θ_1	0.81411	52169.46
Θ_2	0.92002	21063.18
Θ_3	0.93452	17145.29
Θ_4	0.93393	17321.03
$M_{2->1}$	0.87707	33018.07
$M_{3->2}$	0.75283	77826.63
$M_{4->2}$	0.53579	177839.17
Ln[Prob(D|G)]	0.99538	1159.12

Potential Problems

This section reports potential problems with your run, but such reporting is often not very accurate. Whith many parameters in a multilocus analysis, it is very common that some parameters for some loci will not be very informative, triggering suggestions (for example to increase the prior range) that are not sensible. This suggestion tool will improve with time, therefore do not blindly follow its suggestions. If some parameters are flagged, inspect the tables carefully and judge wether an action is required. For example, if you run a Bayesian inference with sequence data, for macroscopic species there is rarely the need to increase the prior for Theta beyond 0.1; but if you use microsatellites it is rather common that your prior distribution for Theta should have a range from 0.0 to 100 or more. With many populations (>3) it is also very common that some migration routes are estimated poorly because the data contains little or no information for that route. Increasing the range will not help in such situations, reducing number of parameters may help in such situations.

No warning was recorded during the run
